# Risk of Postoperative Ischemic Stroke and Myocardial Infarction in Patients Operated for Cancer

**DOI:** 10.1245/s10434-023-14688-6

**Published:** 2023-12-13

**Authors:** Juhana Rautiola, Johan Björklund, Renata Zelic, Gustaf Edgren, Matteo Bottai, Magnus Nilsson, Per Henrik Vincent, Hanna Fredholm, Henrik Falconer, Annika Sjövall, Per J. Nilsson, Peter Wiklund, Markus Aly, Olof Akre

**Affiliations:** 1 Department of Molecular Medicine and Surgery, Karolinska Institutet, Stockholm, Sweden; 2 Department of Pelvic Cancer, Karolinska University Hospital, Stockholm, Sweden; 3 Clinical Epidemiology Division, Department of Medicine, Karolinska Institutet, Stockholm, Sweden; 4 Department of Cardiology, Södersjukhuset, Stockholm, Sweden; 5 Division of Biostatistics, Institute of Environmental Medicine, Karolinska Institutet, Stockholm, Sweden; 6 Division of Surgery and Oncology, Department of Clinical Science, Intervention and Technology, Karolinska Institutet, Stockholm, Sweden; 7 Department of Upper Abdominal Diseases, Karolinska University Hospital, Stockholm, Sweden; 8 Department of Women’s and Children’s Health, Karolinska Institutet, Stockholm, Sweden; 9 Department of Urology, Icahn School of Medicine at Mount Sinai, New York, USA

**Keywords:** Oncological surgery, Arterial ischemic events, Ischemic stroke, Myocardial infarction

## Abstract

**Background:**

Risk assessment for ischemic stroke (IS) and myocardial infarction (MI) is done routinely before surgery, but the increase in risks associated with surgery is not known. The aim of this study is to assess the risk of arterial ischemic events during the first year after oncological surgery.

**Methods:**

We used Swedish healthcare databases to identify 443,300 patients who underwent cancer surgery between 1987 and 2016 and 4,127,761 matched comparison subjects. We estimated odds ratios (ORs) for myocardial infarction and ischemic stroke during the hospitalization with logistic regression and calculated 1-year cumulative incidences and hazard ratios (HRs) with 95% confidence intervals (CIs) for the outcomes after discharge.

**Results:**

The cumulative incidences of myocardial infarction and ischemic stroke during the first postoperative year were 1.33% and 1.25%, respectively. In the comparison cohort, the corresponding 1-year cumulative incidences were 1.04% and 1.00%. During the hospitalization, the OR for myocardial infarction was 8.81 (95% CI 8.24–9.42) and the OR for ischemic stroke was 6.71 (95% CI 6.22–7.23). After discharge, the average HR during follow-up for 365 days was 0.90 (95% CI 0.87–0.93) for myocardial infarction and 1.02 (95% CI 0.99–1.05) for ischemic stroke.

**Conclusions:**

We found an overall increased risk of IS and MI during the first year after cancer surgery that was attributable to events occurring during the hospitalization period. After discharge from the hospital, the overall risk of myocardial infarction was lower among the cancer surgery patients than among matched comparison subjects.

**Supplementary Information:**

The online version contains supplementary material available at 10.1245/s10434-023-14688-6.

Arterial ischemic events manifested by ischemic stroke (IS) and myocardial infarction (MI) are a major cause of death. Preoperative risk assessment for these events is done in clinical routine to optimize the patients’ chances of surviving the surgery under the assumption that the stress of the surgical and anesthesiologic trauma triggers these events.^1^ Every year, millions of patients suffer from perioperative myocardial infarction and ischemic stroke, causing significant morbidity.^2–4^ Indeed, cardiovascular disease is the leading cause of death in the postoperative period.^5,6^ There is, however, a paucity of data on the occurrence of postoperative arterial events in relation to the incidence in the background population.

Approximately half of all patients diagnosed with cancer undergo surgery with the goal of achieving radical removal of the tumor,^7^ and the risk of dying from an untreated cancer is generally sufficiently high to justify major surgery without delay even in older patients with comorbidities. Cancer itself may not be a strong risk factor for arterial events.^8,9^ The different treatments for cancer may, however, be prothrombotic, leading to an increased risk of ischemic heart disease and stroke among cancer patients.^10–12^

We studied the risks of ischemic stroke and myocardial infarction during the first year after major surgery for different types of cancer. Furthermore, we compared the occurrence among these patients with the occurrence in a matched reference cohort to evaluate whether the risks of these outcomes may be increased by surgery, and if so, for how long the potentially increased risk may last.

## Methods

This is a nationwide, retrospective cohort study of patients who were operated on for cancer and a matched comparison population followed up for the occurrence of ischemic stroke or myocardial infarction.

### Study Population

The cancer surgery cohort consisted of all patients who were diagnosed with cancer of the breast, urinary bladder, colon or rectum, esophagus, gallbladder, gynecological organs, stomach, kidney or upper urothelial, lung, pancreas, and prostate from 1987 to 2016, and who had a related major surgical procedure code in the Swedish National Patient Register (established in 1964, with full national coverage of inpatient care in 1987 and of outpatient care from 1997 with nationwide coverage from 2001).^13^ To identify diagnostic codes, we used the International Classification of Diseases version 9 (ICD-9) from 1987 to 1996 and version 10 (ICD-10) from 1997 onward. For surgical procedures between 1987 and 1996, we used national classification for operations^14^ and, from 1997 onward, a nationally adapted version of the Nordic Medico-Statistical Committee (NOMESCO) Classification of Surgical Procedures.^15^ The specific inclusion codes for each diagnosis and the connected procedures are presented in Supplementary Table 1. Start of follow-up was the date of the index cancer surgery, defined as the date of admission for the surgery. We also retrieved all other diagnoses and procedures for the year preceding and for the first year following the index date.

### Comparison Cohort

For each patient in the cancer surgery cohort, we selected 10 comparison subjects matched by year of birth, sex, and county of residence in a randomized fashion from the Swedish Total Population Register. Comparison subjects were assigned an index date corresponding to the index date of their respective cancer surgery patient. Through linkage with the Cancer Register, we ascertained that the comparison subjects were cancer-free before the index date.

### Outcome Definition and Follow-Up

All cancer surgery patients and control persons were followed up using the Cause-of-Death Register (established in 1950) and the Swedish Patient Register for the occurrence of a fatal or non-fatal ischemic stroke or myocardial infarction within 1 year from the index date. Myocardial infarction was identified by the following codes: 410, 423, and 429 (ICD-9) and I21-I23 (ICD-10). Ischemic stroke was identified by: 433, 434, and 436 (ICD-9) and I63 or I64.9 (ICD-10). The sensitivity of the Patient Register is high for both myocardial infarction and cerebral ischemic stroke; in validation studies, sensitivities for myocardial infarction and ischemic stroke were 91.5% and 96%, respectively.^13,16^

Since we lacked information on the exact day of the event during the hospitalization period, and given potentially different probabilities of having the outcome during and after the hospitalization, we further separated the outcomes occurring during the index hospitalization from those happening after discharge. For the analysis of outcomes during the index hospitalization, the comparison subjects were assigned the same length of hospitalization as their matched cancer surgery patients. For the analysis of outcomes after the discharge, all subjects were followed until they had an event, died from another cause, or were administratively censored at 1 year from the index date, whichever came first.

### Statistical Analyses

We calculated 1-year cumulative risks of each outcome and 1-year crude odds ratios (ORs) among the cancer surgery patients and the comparison population. For analysis of the outcomes during hospitalization, we used logistic regression to estimate ORs and 95% confidence intervals (CIs). For outcomes after the discharge, we estimated the cumulative incidence function of the outcome using the Aalen–Johansen estimator, with death from other causes treated as a competing event.^17^ The HRs and 95% CIs were estimated using flexible parametric models,^18^ with time since the discharge as the underlying time scale (modeled with four degrees of freedom). We evaluated the proportional hazards assumption by testing the null hypothesis of zero slope of the scaled Schoenfeld residuals.^19^ Since we were interested in the change in the HR over the follow-up time, even though the proportional hazards assumption was not violated for all cancer cohorts, we allowed the effect of exposure to vary over the follow-up time (modeled with three degrees of freedom). All analyses were done adjusting for the matching variables.^20^ Furthermore, previous outcome and comorbidities (hypertension, diabetes mellitus, congestive heart failure, renal disease, chronic pulmonary disease, valvular disease, cardiac arrhythmia, peripheral vascular disease, and anemia) were a priori identified as potential confounders and were included in all the analyses (Supplementary Tables 2 and 3).

Since the outpatient portion of the Patient Register did not have full nationwide coverage until 2001, we conducted a sensitivity analysis in the population restricted to subjects operated in 2002 or later to allow for a 1-year lookback period before the index date for identification of the baseline covariates. Furthermore, 37% of patients had been operated on for breast cancer, which is often done in day surgery and is less traumatic compared with other included surgeries. We therefore repeated all analyses excluding the breast cancer patients and comparison subjects.

All analyses were conducted using Stata version 16.1 and 17.0. Our study was approved by the Regional Ethics Committee in Stockholm (DNR: 2017/936-31). Informed consents were not collected, and the data used for analyses were pseudonymized.

## Results

After excluding patients not fulfilling the study criteria, a total of 443,300 cancer patients and 4,127,761 matched comparison subjects remained in the analysis (flowchart shown in Supplementary Fig. 1). The baseline characteristics and the crude proportions of subjects with an outcome during follow-up are presented in Table 1, and cancer-specific characteristics are presented in Supplementary Table 4.

**Table 1:** Baseline characteristics of patients undergoing cancer surgery and matched comparison population

	Number (%)	
	Cancer surgery population (*n* = 443,300)	Comparison population (*n* = 4,127,760)
Cancer type		
Bladder	8630 (1.95)	79,040 (1.91)
Breast	163,799 (36.95)	1,547,599 (37.49)
Colorectal	115,235 (25.99)	1,045,758 (25.33)
Gallbladder	2267 (0.51)	20,957 (0.51)
Gastroesophageal	15,299 (3.45)	140,769 (3.41)
Gynecological	58,469 (13.19)	550,587 (13.34)
Kidney and UTUC	21,297 (4.80)	197,263 (4.78)
Lung	14,111 (3.18)	131,523 (3.19)
Pancreatic	4040 (0.91)	37,637 (0.91)
Prostate	40,153 (9.06)	376,628 (9.12)
Age (median, IQR)	67 (58, 75)	66 (57, 74)
Age groups (years)		
≤ 49	48,384 (10.91)	477,654 (11.57)
50–59	77,881 (17.57)	756,029 (18.32)
60–69	133,801 (30.18)	1,260,366 (30.53)
70–79	119,317 (26.92)	1 077 882 (26.11)
≥ 80	63,917 (14.42)	555,830 (13.47)
Women	290,713 (68.21)	2,729,277 (68.79)
Length of hospitalization (days, median; IQR)	6 (2.00, 10.00)	
Outcome		
Myocardial infarction, in total	5903 (1.33)	43,023 (1.04)
During hospitalization	1855 (0.42)	1785 (0.04)
After discharge	4048 (0.91)	41,238 (1.00)
Ischemic stroke, in total	5532 (1.25)	41,214 (1.00)
During hospitalization	1256 (0.28)	1744 (0.04)
After discharge	4276 (0.96)	39,470 (0.96)
Ischemic heart disease	14,741 (3.46)	96,460 (2.43)
History of myocardial infarction	3289 (0.74)	26,728 (0.65)
Congestive heart failure	8073 (1.82)	57,723 (1.40)
Cardiac arrhythmia	16,333 (3.68)	99,605 (2.41)
Valvular disease	3479 (0.78)	20,568 (0.50)
Peripheral vascular disease	3452 (0.78)	22,178 (0.54)
Hypertension	33,604 (7.58)	149,432 (3.62)
Cerebrovascular disease	6986 (1.58)	60,585 (1.47)
History of ischemic stroke	4174 (0.94)	37,848 (0.92)
TIA	1539 (0.35)	12,243 (0.30)
Chronic pulmonary disease	10,175 (2.30)	55,293 (1.34)
Diabetes mellitus	17,436 (3.93)	100,612 (2.44)
Renal disease	2357 (0.53)	15,740 (0.38)
Anemia	10,615 (2.39)	10,463 (0.25)
All-cause mortality, at 90 days	12,773 (2.88)	22,402 (0.54)
All-cause mortality, at 1 year	43,025 (9.71)	94,911 (2.30)

*IQR* interquartile range, *TIA* transient ischemic attack, *UTUC* upper tract urothelial cancer

The overall crude cumulative incidences for myocardial infarction and ischemic stroke during the first year after cancer surgery for the entire cohort of cancer patients were 1.33% and 1.25%, respectively. The corresponding risks in the comparison population were 1.04% and 1.00% (Table 2). The crude ORs for the association between surgery for any malignancy and risk of myocardial infarction or stroke during the first year after surgery were 1.28 (95% CI 1.25–1.32) and 1.25 (95% CI 1.22–1.29), compared with the background population. The ORs varied between different types of cancer (Supplementary Table 5). During 1-year follow-up, matched subjects had more severe forms of myocardial infarction recorded as percutaneous coronary intervention (PCI) or cardiac bypasses during the same hospitalization than for MI. However, this difference was mostly due to the MIs recorded during hospitalization for cancer surgery. When looking at outcomes after the discharge from hospital, similar proportions of severe or lethal myocardial infarctions were recorded (Supplementary Table 6).

**Table 2:** Absolute risks for arterial ischemic events during the first year of follow-up

Cancer type	Absolute risk		Risk difference (%)	95% CI
	Cancer surgery population (%)	Comparison population (%)		
All cancers				
MI	1.33	1.04	0.29	0.25, 0.32
IS	1.25	1.00	0.25	0.22, 0.28
Bladder				
MI	2.19	1.34	0.85	0.54, 1.17
IS	1.63	1.13	0.51	0.23, 0.78
Breast				
MI	0.68	0.67	0.01	− 0.03, 0.05
IS	0.81	0.74	0.07	0.02, 0.11
Colorectal				
MI	2.33	1.68	0.65	0.56, 0.74
IS	2.02	1.56	0.46	0.37, 0.54
Gallbladder				
MI	1.85	1.31	0.54	−0.04, 1.12
IS	1.85	1.11	0.75	0.17, 1.32
Gastroesophageal				
MI	2.52	1.63	0.88	0.63, 1.14
IS	1.99	1.35	0.64	0.41, 0.87
Gynecological				
MI	0.81	0.70	0.11	0.03, 0.19
IS	1.02	0.74	0.27	0.19, 0.36
Kidney and UTUC				
MI	1.77	1.18	0.59	0.41, 0.78
IS	1.45	1.08	0.37	0.20, 0.54
Lung				
MI	2.04	1.02	1.02	0.78, 1.26
IS	1.62	0.86	0.75	0.54, 0.97
Pancreatic				
MI	1.86	0.90	0.96	0.53, 1.39
IS	1.83	0.85	0.98	0.55, 1.40
Prostate				
MI	0.67	0.94	− 0.27	− 0.36, − 0.19
IS	0.45	0.71	− 0.26	− 0.33, − 0.19

*MI* myocardial infarction, *IS* ischemic stroke, *UTUC* upper tract urothelial cancer

### Events Occurring during Hospitalization

The median duration of hospitalization was 6 days [interquartile range (IQR), 2–10 days]. During the hospitalization, the odds of arterial events were higher in the surgery cohort than among matched comparison persons. Among all cancer patients, the ORs during hospitalization were 8.81 (95% CI 8.24–9.42) for myocardial infarction and 6.71 (95% CI 6.22–7.23) for ischemic stroke. The ORs for MI and IS in patients operated on for prostate cancer were 4.64 (95% CI 3.00–7.19) and 3.54 (95% CI 2.08–6.03), whereas the corresponding ORs for pancreatic cancer were 13.61 (95% CI 8.96–20.68) and 13.56 (95% CI 8.94–20.58). The specific ORs during hospitalization are presented for all cancer forms in Table 3.

**Table 3:** Risk of arterial ischemic events in patients with cancer surgery during hospitalization

Cancer type	Myocardial infarction		Ischemic stroke	
	OR	95 % CIs	OR	95 % CIs
Bladder	7.51	5.35, 10.53	5.21	3.50, 7.76
Breast	5.25	4.11, 6.70	6.65	5.36, 8.24
Colorectal	9.68	8.84, 10.59	6.72	6.06, 7.47
Gallbladder	6.76	3.46, 13.23	7.27	2.85, 18.54
Gastroesophageal	9.01	7.25, 11.18	6.43	4.99, 8.29
Gynecological	7.58	5.89, 9.74	8.38	6.56, 10.72
Kidney and UTUC	10.24	7.63, 13.74	5.73	4.22, 7.76
Lung	13.61	8.96, 20.68	13.56	8.94, 20.58
Pancreatic	14.51	7.67, 27.45	4.49	2.04, 9.86
Prostate	4.64	3.00, 7.19	3.54	2.08, 6.03
All cancers	8.81	8.24, 9.42	6.71	6.22, 7.23
Women	0.40	0.37, 0.43	0.59	0.55, 0.64
Age groups (years)				
≤ 49	0.14	0.09, 0.22	0.12	0.07, 0.21
50–59	0.38	0.31, 0.48	0.47	0.38, 0.58
60–69	1.00	–	1.00	–
70–79	2.64	2.39, 2.91	2.74	2.45, 3.07
≥ 80	5.22	4.72, 5.77	5.25	4.67, 5.89

*MI* myocardial infarction, *IS* ischemic stroke, *UTUC* upper tract urothelial cancer

### Acute Myocardial Infarction after Discharge

**Fig. 1:**
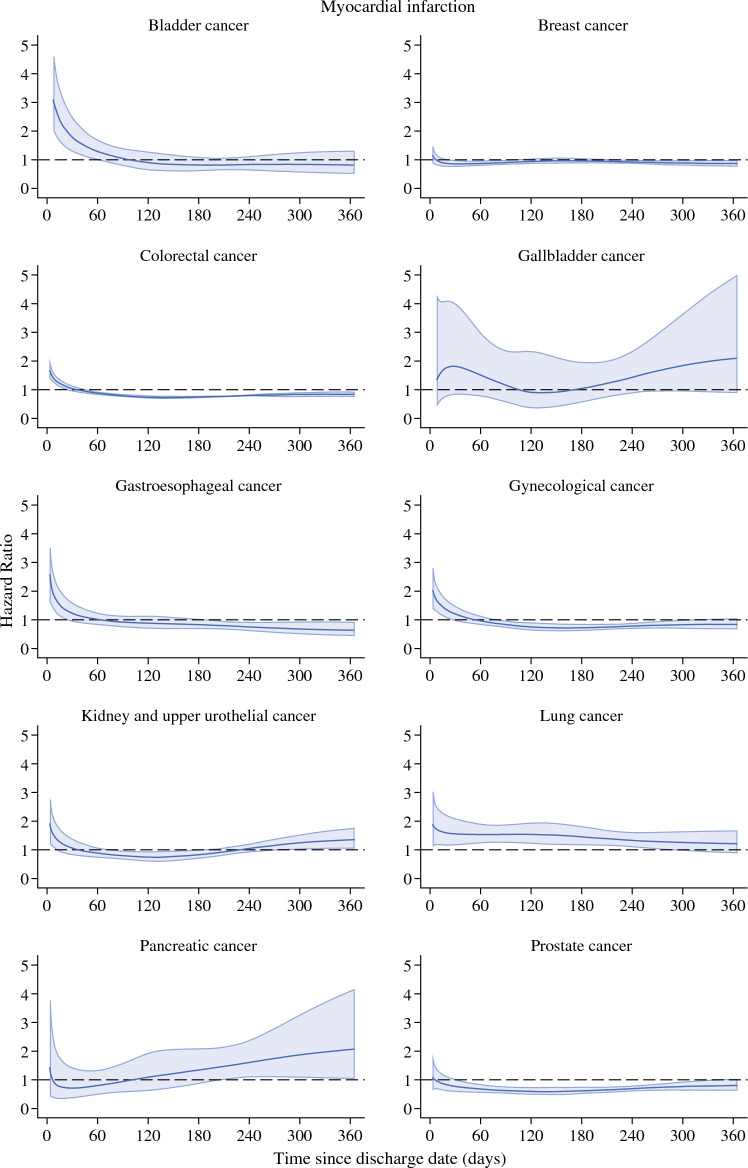
Hazard ratios for myocardial infarction in different cancer populations. The figure presents hazard ratios for myocardial infarction the first year after cancer surgery for different cancer types after discharge from the hospital in relation to matched comparator subjects

The hazard among all cancer patients was lower than in the background population 1 year after discharge. For myocardial infarction, the HR at 1 year from discharge for all-cancer population was 0.90 (95% CI 0.87–0.93; Supplementary Fig. 2 and Supplementary Table 4). However, the hazards varied between cancers; while patients operated on for cancers in the urinary bladder or lung had higher rates of myocardial infarction than the comparison population, patients operated on for cancers in the breast, colon/rectum, gynecological organs, or prostate had lower hazard (Fig. 1). After peaking during the hospitalization period, the hazard of myocardial infarction among cancer patients decreased, for some cancers, even below the hazard of the comparison population (Fig. 1; Table 4). For example, the HR at 30 days after discharge for patients who had had surgery of the gynecological organs was 1.26 (95% CI 1.01–1.57) and at 90 days was 0.87 (95% CI 0.74–1.03). Furthermore, the crude 1-year cumulative incidence of myocardial infarction after discharge from hospital in the prostate cancer surgery patients was 0.59% (95% CI 0.52–0.67) as compared with 0.92% (95% CI 0.89–0.95) among the comparison population. The corresponding number for lung cancer patients were 0.98% (95% CI 0.93–1.04) and 1.58% (95% CI 1.38–1.80). The cumulative incidences for other tumor forms are shown in Supplementary Fig. 3.

**Table 4:** Risk of arterial ischemic events in patients with cancer surgery after the discharge

Cancer type	Myocardial infarction		Ischemic stroke	
	HR	95% CIs	HR	95% CIs
Bladder				
Average	1.11	0.91, 1.35	1.21	0.98, 1.48
30 days	1.98	1.41, 2.78	1.80	1.21, 2.68
90 days	1.16	0.86, 1.55	1.37	1.02, 1.83
1 year	0.90	0.55, 1.45	0.84	0.50, 1.43
Breast				
Average	0.92	0.86, 0.98	0.98	0.92, 1.04
30 days	0.85	0.73, 0.99	0.96	0.84, 1.09
90 days	0.90	0.82, 0.99	0.98	0.90, 1.07
1 year	0.87	0.75, 1.00	0.88	0.77, 1.01
Colorectal				
Average	0.86	0.81, 0.90	1.01	0.96, 1.07
30 days	1.13	1.01, 1.25	1.22	1.10, 1.35
90 days	0.86	0.79, 0.93	1.04	0.96, 1.12
1 year	0.91	0.81, 1.03	0.89	0.79, 1.00
Gallbladder				
Average	1.40	0.93, 2.11	1.77	1.21, 2.59
30 days	1.94	0.87, 4.29	1.96	1.01, 3.81
90 days	1.25	0.61, 2.55	2.14	1.24, 3.68
1 year	2.28	0.96, 5.41	1.68	0.60, 4.71
Gastroesophageal				
Average	0.90	0.77, 1.05	1.29	1.10, 1.50
30 days	1.26	0.95, 1.67	1.68	1.26, 2.25
90 days	0.95	0.76, 1.20	1.38	1.12, 1.69
1 year	0.69	0.46, 1.04	0.93	0.63, 1.38
Gynecological				
Average	0.88	0.79, 0.98	1.12	1.02, 1.23
30 days	1.26	1.01, 1.57	1.66	1.37, 2.01
90 days	0.87	0.74, 1.03	1.29	1.13, 1.48
1 year	0.88	0.69, 1.12	0.81	0.64, 1.02
Kidney and UTUC				
Average	1.01	0.89, 1.15	1.07	0.93, 1.22
30 days	1.21	0.92, 1.59	1.31	0.98, 1.74
90 days	0.91	0.74, 1.11	1.15	0.95, 1.39
1 year	1.53	1.17, 2.00	1.05	0.77, 1.43
Lung				
Average	1.44	1.27, 1.67	1.41	1.19, 1.66
30 days	1.80	1.34, 2.43	2.31	1.68, 3.18
90 days	1.77	1.44, 2.17	1.49	1.16, 1.91
1 year	1.41	1.02, 1.95	1.14	0.78, 1.67
Pancreatic				
Average	1.28	0.91, 1.79	2.32	1.75, 3.09
30 days	0.82	0.41, 1.66	1.74	0.91, 3.34
90 days	1.09	0.65, 1.82	1.90	1.17, 3.07
1 year	2.39	1.20, 4.78	3.82	2.05, 7.13
Prostate				
Average	0.70	0.61, 0.80	0.67	0.57, 0.79
30 days	0.70	0.52, 0.93	0.67	0.49, 0.93
90 days	0.56	0.46, 0.69	0.56	0.45, 0.71
1 year	0.73	0.56, 0.95	0.52	0.36, 0.75
All cancers				
Average	0.90	0.87, 0.93	1.02	0.99, 1.05
30 days	1.09	1.02, 1.17	1.22	1.14, 1.30
90 days	0.89	0.85, 0.94	1.06	1.02, 1.11
1 year	0.92	0.86, 0.99	0.88	0.82, 0.95
Women	0.57	0.56, 0.58	0.78	0.77, 0.80
Age groups (years)				
≤ 49	0.11	0.09, 0.12	0.11	0.10, 0.12
50–59	0.42	0.40, 0.44	0.38	0.36, 0.40
60–69	1.00	–	1.00	–
70–79	2.05	2.00, 2.10	2.40	2.34, 2.47
≥ 80	3.89	3.79, 3.99	4.96	4.82, 5.10

*MI* myocardial infarction, *IS* ischemic stroke, *UTUC* upper tract urothelial cancer

### Ischemic Stroke after Discharge

**Fig. 2:**
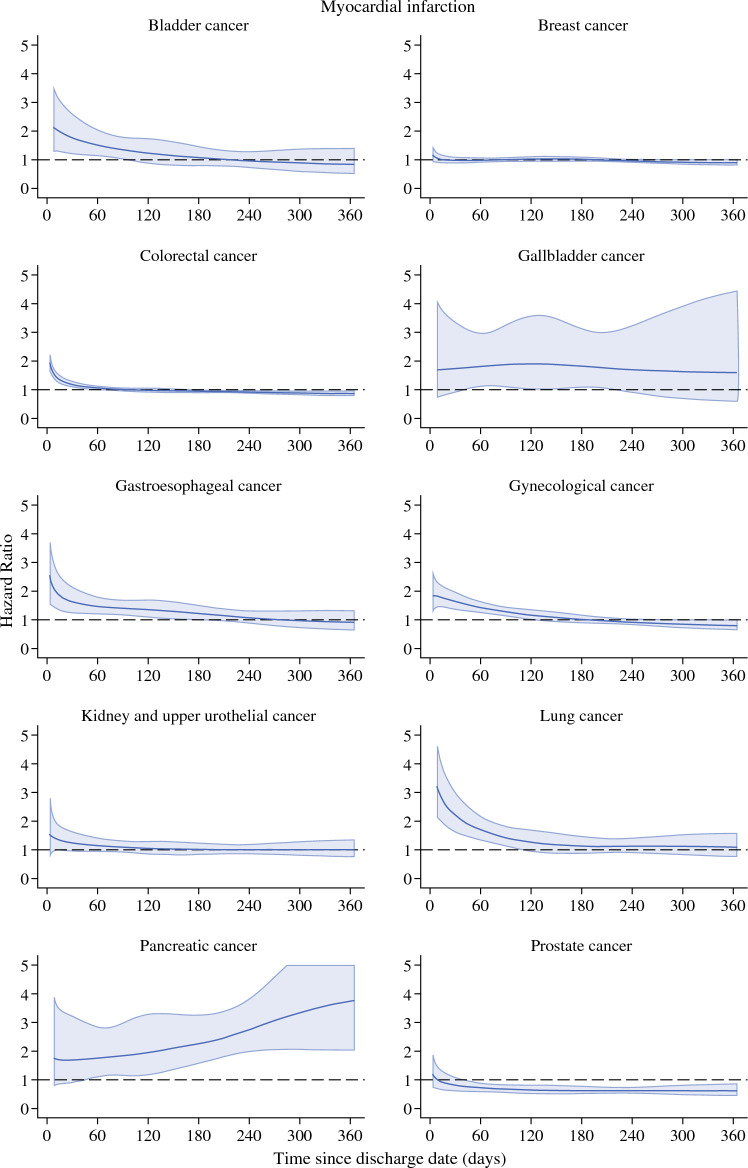
Hazard ratios for ischemic stroke in different cancer populations. The figure presents hazard ratios for ischemic stroke the first year after cancer surgery for different cancer types after discharge from the hospital in relation to matched comparator subjects

The HR at 1 year from discharge for ischemic stroke for the all-cancer population was 1.02 (95% CI 0.99–1.05; Table 4). The increased hazard of stroke in the cancer surgery group persisted longer during follow-up period than for myocardial infarction (Supplementary Fig. 2). For example, among patients who had undergone surgery of the gynecological organs, the HRs at 30 and 90 days were 1.66 (95% CI 1.37–2.01) and 1.29 (95% CI 1.13–1.49), respectively (Fig. 2 and Table 4). The crude 1-year cumulative incidence after discharge of ischemic stroke in the prostate cancer surgery patients was 0.40% (95% CI 0.34–0.46) as compared with 0.69% (95% CI 0.66–0.71) among the comparison population. The corresponding numbers for lung cancer patients were 0.83% (95% CI 0.78–0.88) and 1.19% (95% CI 1.02–1.38). The cumulative incidences for other tumor forms are shown in Supplementary Fig. 4.

### Age and Sex

In cancers affecting both sexes, females had a lower risk of events than men, with HRs of 0.57 (95% CI 0.56–0.58) for myocardial infarction and 0.78 (95% CI 0.77–0.80) for ischemic stroke. The risks of both outcomes increased strongly with increasing age. The HR for patients up to 49 years of age was 0.11 (95% CI 0.10–0.12) for both myocardial infarction and stroke, compared with patients who were 60–69 years of age at the time of surgery (Table 4).

### Sensitivity Analyses

Restricting the analysis to subjects operated on in 2002 or later, as well as excluding the patients with breast cancer, resulted only in marginal changes of the results (Supplementary Tables 7, 8, 9, 10 and Supplementary Figs. 3, 4).

## Discussion

In this population-based nationwide study of over 400,000 patients who had undergone cancer surgery, we found a crude 1-year absolute risk of postoperative myocardial infarction and ischemic stroke of 1.33% and 1.25% after surgery, an absolute excess risk of less than 0.3% compared with the matched comparison population unexposed to surgery. There was, however, considerable variation of absolute excess risks between cancer types, ranging from no excess risk to around 1% during the first postoperative year. The excess occurrence of myocardial infarction and ischemic stroke was mostly attributable to events occurring early in the postoperative care after the index surgery. After discharge from hospital, there was in general little or no excess risk of infarction or stroke associated with cancer surgery. For myocardial infarction, the overall risk was even decreased after discharge. Our data suggest that surgery itself causes a trigger effect leading to an excess number of events mainly during the hospitalization. The duration of the initial transient increase in incidence was longer for ischemic stroke than for myocardial infarction. As with the general occurrence patterns of arterial thromboembolic or atherosclerotic disease, increasing age and male sex were both strongly associated with increased risk.

Patients with cancer in the prostate, breast, or gynecological organs had the lowest incidence of both outcomes. Unlike the other tumor forms, prostate cancer surgery was associated with a markedly reduced incidence of stroke and myocardial infarction, probably reflecting the selection of healthy men, first through the mode of detection with PSA testing, then through selection to surgical treatment, and finally perhaps through the relative rarity of advanced disease at the time of diagnosis, which otherwise might be a strong risk factor for adverse vascular outcomes. In contrast, patients with lung cancer had a significantly increased risk of both outcomes, likely attributable to a higher smoking prevalence and generally poorer health status in this patient group rather than to the surgery itself. Gallbladder and pancreatic cancer had elevated risks, but the disease aggressiveness and high mortality in those cancer types led to more variable and imprecise risk estimates than for the other disease cohorts. The large variation in risk across categories of tumors, age, and sex groups may motivate more individualized preoperative risk assessment than is commonplace today.

There are several plausible pathophysiological mechanisms for an increased risk of stroke and myocardial infarction due to surgery. The surgical trauma may lead to hypercoagulability, inflammation, stress, and catabolic states, which all may trigger and increase risk for vascular complications.^21,22^ Further, anesthesia during surgery may lead to both transient hypotension and coagulopathy, triggering both cardiac complications and ischemic stroke.^23^ The lack of a notably increased risk associated with cancer surgery in addition to the time dynamic of elevated hazard ratios during early follow-up with reduced risks thereafter indicate that the cancer surgery is primarily triggering an already prevalent thrombogenic process.

Patients diagnosed with cancer have been reported to have an elevated risk of arterial thromboembolism. Navi et al.^12^ found an incidence of 4.7% in all cancer patients as compared with 2.2% in a matched cohort during the first 6 months after diagnosis. We found a considerably lower overall risk that mostly did not differ between the surgery patients and the matched comparison population. These inconsistencies may be explained by differences in the selection of study populations. Our study population was nationwide and population based, whereas Navi et al. identified their exposed cohort through the Surveillance, Epidemiology, and End Results (SEER) database, and their comparison cohort through the Medicare registry with patients above 65 years of age. Furthermore, they also included patients with advanced disease and thus patients receiving oncological treatments, which may increase the incidence of outcomes. In our data, we lack information on adjuvant chemotherapy and radiotherapy during the study period and cannot separate the effect of surgery from the potential effect of other treatments. Some studies indicate that cytotoxic drugs and radiotherapy may cause damage to the vascular endothelium and activate the coagulation cascade, leading to arterial thromboembolism or atherosclerotic events.^24–29^

The infrastructure of the Swedish population and healthcare databases allowed us to identify, include, and follow more than 400,000 cancer surgery patients and over 4,000,000 randomly selected comparison persons. Still, using large population registers entails several limitations. First, we lack detailed information on disease severity as well as important exposures such as tobacco smoking, and nuances of other cancer treatments. Second, the outcome ascertainment may entail some degree of misclassification, and although both patients with ischemic stroke and those with myocardial infarction are nearly always hospitalized, there may be unreported events, especially with respect to myocardial infarction, where less severe events may go undiagnosed. That said, although the diagnostic intensity may have been higher among the cancer surgery patients than in the comparison population, it is likely that underdiagnosis of both outcomes is so uncommon that it is an improbable explanation of the observed effects, at least outside of the index hospitalization. Indeed, when finding records with PCI or cardiac bypass codes alongside with myocardial infarction, for most cancers, a higher proportion of comparators had lethal or severe outcome, indicating that cancer surgery patients are monitored closely postoperatively. Nevertheless, it seems that this difference is mostly due to outcomes identified during hospitalization for cancer surgery. When looking only at the outcomes identified after the discharge from the hospital, we observe very similar proportions of lethal outcomes among cancer surgery patients and comparators. Third, although we could assess comorbidities through inpatient and outpatient diagnoses, the limitations of the registration of chronic diseases that do not always lead to hospitalization, such as diabetes, kidney disease, and hypertension, leads to misclassification of comorbidities in our data. This, and the lack of information on the severity of the comorbidities, may have affected the adjusted estimates.

To our knowledge, this is the first study to date to investigate the difference in incidence of ischemic stroke and myocardial infarction between patients undergoing cancer surgery and the general population. We found a somewhat increased risk of these events during hospitalization that was mainly attributable to the period of hospitalization after surgery, which may indicate that the surgical trauma triggers events of arterial thromboembolism. The main clinical implication of the study is the absolute and relative estimates of risks and the distribution of events over time after surgery. Furthermore, we found substantial difference in the risk of these events for different surgical procedures or tumor forms, which may suggest more individualized risk assessment. Further studies are needed to identify patients at high risk for arterial ischemic events after cancer surgery and the potential need for preventive medication. Cardiovascular and cerebrovascular events will remain a considerable problem in the postoperative period, but our data suggest that the long-term risk is not considerably increased by the surgery itself.

### Supplementary Information

Below is the link to the electronic supplementary material.

Supplementary file1 (DOCX 2127 kb)
