# Supplementary material for: Risk of Postoperative Ischemic Stroke and Myocardial Infarction in Patients Operated for Cancer
Source: Ann Surg Oncol. 2023 Dec 13;31(3):1739–48. doi: 10.1245/s10434-023-14688-6 (PMC10838243; doi:10.1245/s10434-023-14688-6)
Supplement: Supplementary file 1 — Supplementary file1 (DOCX 2127 kb) [file 10434_2023_14688_MOESM1_ESM.docx]

# Supplementary Appendix

# Table of contents

Table 1. Inclusion diagnostic and procedural codes from the Swedish Patient Register 2

Table 2. Full model estimates for the analysis of myocardial infarction during hospitalization (OR) and after discharge (HR), all cancers combined 3

Table 3. Full model estimates for the analysis of ischemic stroke during hospitalization (OR) and after discharge (HR), all cancers combined 4

Figure 1. Flow chart of the patient selection 5

Table 4. Patient characteristics for separate cancer types 7

Table 5. Crude ORs of arterial ischemic events during the first year of follow up 8

Figure 2. Hazard ratios for myocardial infarction and ischemic stroke after discharge from the hospital 9

Figure 3. Cumulative incidence function for myocardial infarction after discharge from the hospital 10

Figure 4. Cumulative incidence function for ischemic stroke after discharge from the hospital 11

Table 6. Lethal or severe myocardial infarctions during the first postoperative year 12

Table 7. Baseline characteristics of cancer surgery population and comparison population, sensitivity analysis of patients recruited since 2002 14

Table 8. Absolute risks for arterial ischemic events during the first year of follow up, sensitivity analysis of patients recruited since 2002. 15

Table 9. Risk of arterial ischemic events during hospitalization in cancer surgery population compared to matched comparison population, sensitivity analysis of patients recruited since 2002 16

Table 10. Risk of arterial ischemic events after discharge from the hospital in the cancer surgery population compared to comparison population, sensitivity analysis of patients recruited since 2002. 17

Figure 5. Cumulative incidence of myocardial infarction and ischemic stroke after discharge from the hospital, patients after 2002 19

Figure 6. Hazard ratios for myocardial infarction and ischemic stroke after discharge from the hospital, patients after 2002 20

Figure legends 21

## Table 1. Inclusion diagnostic and procedural codes from the Swedish Patient Register

| Cancer type | ICD codes^1^ | | Procedural codes | |
| --- | --- | --- | --- | --- |
|  | ICD-9 | ICD-10 | K06 | KVÅ |
| Bladder | 188x | C67x | 6318, 6320–28, 6331 | KCC00, KCC10, KCC20, KCC30, KCC96 |
| Breast | 174x, 175 | C50x | 3801–02, 3810-14, 3816-17, 3820-21, 3830-32, 3840, 3890-91 | HAB00, HAB20, HAB30, HAB40, HAB99, HAC10, HAC15, HAC20, HAC22, HAC25, HAC30, HAC99 |
| Colorectal | 153x, 154x | C18x, C19x, C20x | 4640-44, 4648-54, 4810, 4820-23, 4828-29 | JFA83, JFB20-21, JFB30-31, JFB33-34, JFB40-41, JFB43-44, JFB46-47, JFB50-51, JFB53-54, JFB60-61, JFB63-64, JFB96-97, JGB00-01, JGB03-04, JGB10-11, JGB20, JGB30-31, JGB33-34, JGB36, JGB40, JGB60-61, JGB96-97, JFH00-01, JFH10-11, JFH20, JFH30-31, JFH33, JFH40, JFH96 |
| Gallbladder | 156x | C23, C24x | 5310-13, 5319, 5350-53, 5356-57, 5359, 5398-99 | JKA20-21, JKA96-97, JKC00-01, JKC10, JKC20, JKC30, JKC40, JKC50, JKC96-97, JKW96-98 |
| Gastroesophageal | 150x, 151x | C15x, C16x | 2820-22, 2829, 4411-26, 4429-30, 4432, 4434-35, 4439 | JCC00, JCC10-12, JCC20, JCC30, JCC96-97, JDC00, JDC10-11, JDC20, JDC30, JDC40, JDC96-97, JDD00-01, JDD96 |
| Gynecological | 179x, 180x, 182x, 183x, 184x | C53x, C54x, C55, C56, C57x | 7010-12,7020-23, 7030-33, 7120-21, 7123, 7210-11, 7214-18, 7220-23, 7228-29, 7240, 7249-52, 7259-63, 7269, 7310-11 | LAD00-01, LAE10-11, LAE20-21, LAF00-01, LAF10-11, LAF20, LAF30, LDB00, LBE00-01, LBE03, LCB97-98, LCC00-01, LCC05, LCC10-11, LCC20, LCC96-97, LCD00-01, LCD04, LCD10-11, LCD30-31, LCD40, LCD96-97, LCE00, LCE10, LCE20, LCE96, LCF00-01, LCF96-97, LDC10, LDC96 |
| Kidney and UTUC | 189x | C64, C65, C66 | 6020, 6030, 6040-43 | KAC00-01, KAC20-21, KAD00-01, KAD10, KAD40, KAD51-52, KAD56, KAD60, KAD96-98, KBC00, KBD00, KBD22 |
| Lung | 162x | C34x | 3520, 3530, 3532, 3534-35, 3539-43 | GDB00-01, GDB10-11, GDB20-21, GDB96-97, GDC00-01, GDC10-11, GDC13, GDC20, GDC23, GDC26, GDC96-97, GDD00-01, GDD10-11, GDD20, GDD23, GDD26, GDD96-97 |
| Pancreas | 157x | C25x | 5510-17, 5519 | JLC00, JLC10-11, JLC20, JLC30, JLC40, JLC50, JLC96 |
| Prostate | 185 | C61 | 6611, 6631, 6633, 6639 | KEC00-01, KEC10, KEC20 |
| Testicular | 186x | C62x | 6740-42, 6751-52, 6120 | KFC00, KFC10, KFC15, KFC96, KFD00 |
| Abbreviations: ICD, International Classification of Disease (Swedish version); K06, The national classification for operations, sixth edition (1988); KVÅ, Klassifikation av vårdåtgärder, an adapted version of NOMESCO classification of surgical procedures  ^1^ICD-9 and ICD-10 codes recorded as a main, or any of the 30 contributory diagnoses | | | | |

## Table 2. Full model estimates for the analysis of myocardial infarction during hospitalization (OR) and after discharge (HR), all cancers combined

|  | Myocardial infarction | | | |
| --- | --- | --- | --- | --- |
|  | during hospitalization | | after the discharge | |
|  | OR | 95 % CIs | HR | 95 % CIs |
| Cancer surgery | 8.81 | 8.24, 9.42 | 0.90 | 0.87, 0.93 |
| Ischemic heart disease | 6.36 | 5.78, 7.01 | 3.30 | 3.19, 3.40 |
| Ischemic stroke | 2.00 | 1.71, 2.34 | 1.32 | 1.25, 1.40 |
| Hypertension | 0.84 | 0.75, 0.93 | 0.89 | 0.86, 0.92 |
| Diabetes mellitus | 1.38 | 1.23, 1.55 | 1.88 | 1.81, 1.94 |
| Congestive heart failure | 1.81 | 1.60, 2.04 | 1.82 | 1.75, 1.90 |
| Renal disease | 1.38 | 1.11, 1.73 | 1.65 | 1.54, 1.77 |
| Chronic pulmonary disease | 0.97 | 0.82, 1.14 | 1.42 | 1.35, 1.49 |
| Valvular disease | 1.03 | 0.83, 1.28 | 1.32 | 1.24, 1.41 |
| Cardiac arrhythmia | 0.66 | 0.58, 0.75 | 0.86 | 0.83, 0.90 |
| Peripheral vascular disease | 1.67 | 1.39, 2.01 | 1.59 | 1.50, 1.69 |
| Anemia | 0.94 | 0.79, 1.13 | 1.05 | 0.97, 1.15 |
| Sex | 1.00 | - | 1.00 | - |
| Male |  |  |  |  |
| Female | 0.40 | 0.37, 0.43 | 0.57 | 0.56, 0.58 |
| Age |  |  |  |  |
| ≤49 | 0.14 | 0.09, 0.22 | 0.11 | 0.09, 0.12 |
| 50-59 | 0.38 | 0.31, 0.48 | 0.42 | 0.40, 0.44 |
| 60-69 | 1.00 | - | 1.00 | - |
| 70-79 | 2.64 | 2.39, 2.91 | 2.05 | 2.00, 2.10 |
| ≥80 | 5.22 | 4.72, 5.77 | 3.89 | 3.79, 3.99 |
| Healthcare region* |  |  |  |  |
| 1 | 1.07 | 0.96, 1.20 | 1.21 | 1.17, 1.25 |
| 2 | 0.99 | 0.89, 1.10 | 1.14 | 1.11, 1.18 |
| 3 | 1.18 | 1.06, 1.33 | 1.17 | 1.13, 1.21 |
| 4 | 1.00 | - | 1.00 | - |
| 5 | 1.02 | 0.92, 1.14 | 1.19 | 1.16, 1.23 |
| 6 | 1.03 | 0.91, 1.18 | 1.18 | 1.13, 1.22 |
| Abbreviations: MI, Myocardial infarction; OR, Odds ratio; HR, Hazard ratio; CIs, Confidence intervals  * County of residence was gruped into 6 healthcare regions (Region 1: Skåne (Malmöhus and Kristianstads), Blekinge; Region 2: Halland, Västra Götaland (Älvsborg and Skaraborg), Värmland; Region 3: Kronoberg, Kalmar, Jönköping, Östergötland; Region 4: Stockholm, Gotland; Region 5: Uppsala, Sörmland, Västmanland, Dalarna (Kopparberg), Gävleborg, Örebro; Region 6: Jämtland, Västernorrland, Norrbotten, Västerbotten) | | | | |

## Table 3. Full model estimates for the analysis of ischemic stroke during hospitalization (OR) and after discharge (HR), all cancers combined

|  | Ischemic stroke | | | |
| --- | --- | --- | --- | --- |
|  | during hospitalization | | after the discharge | |
|  | OR | 95 % CIs | HR | 95 % CIs |
| Cancer surgery | 6.71 | 6.22, 7.23 | 1.02 | 0.99, 1.05 |
| Ischemic heart disease | 1.19 | 1.04, 1.36 | 1.15 | 1.10, 1.19 |
| Ischemic stroke | 22.39 | 20.21, 24.80 | 8.07 | 7.82, 8.33 |
| Hypertension | 1.00 | 0.89, 1.12 | 1.11 | 1.07, 1.14 |
| Diabetes mellitus | 1.31 | 1.15, 1.49 | 1.64 | 1.59, 1.71 |
| Congestive heart failure | 1.29 | 1.10, 1.51 | 1.25 | 1.19, 1.31 |
| Renal disease | 0.95 | 0.69, 1.31 | 1.18 | 1.08, 1.28 |
| Chronic pulmonary disease | 0.96 | 0.78, 1.17 | 1.04 | 0.98, 1.10 |
| Valvular disease | 0.87 | 0.65, 1.17 | 1.09 | 1.01, 1.18 |
| Cardiac arrhythmia | 1.17 | 1.03, 1.33 | 1.30 | 1.25, 1.35 |
| Peripheral vascular disease | 1.91 | 1.55, 2.34 | 1.58 | 1.48, 1.69 |
| Anemia | 0.80 | 0.63, 1.01 | 1.03 | 0.94, 1.12 |
| Sex |  |  |  |  |
| Male | 1.00 | - | 1.00 | - |
| Female | 0.59 | 0.55, 0.64 | 0.78 | 0.77, 0.80 |
| Age |  |  |  |  |
| ≤49 | 0.12 | 0.07, 0.21 | 0.11 | 0.10, 0.12 |
| 50-59 | 0.47 | 0.38, 0.58 | 0.38 | 0.36, 0.40 |
| 60-69 | 1.00 | - | 1.00 | - |
| 70-79 | 2.74 | 2.45, 3.07 | 2.40 | 2.34, 2.47 |
| ≥80 | 5.25 | 4.67, 5.89 | 4.96 | 4.82, 5.10 |
| Healthcare region* |  |  |  |  |
| 1 | 0.87 | 0.76, 0.98 | 1.03 | 0.99, 1.06 |
| 2 | 0.94 | 0.84, 1.05 | 1.03 | 1.00, 1.06 |
| 3 | 0.93 | 0.82, 1.06 | 1.01 | 0.97, 1.04 |
| 4 | 1.00 | - | 1.00 | - |
| 5 | 1.00 | 0.89, 1.13 | 1.03 | 0.99, 1.06 |
| 6 | 0.98 | 0.85, 1.14 | 1.09 | 1.05, 1.13 |
| Abbreviations: IS, Ischemic stroke; OR, Odds ratio; HR, Hazard ratio; CIs, Confidence intervals  * County of residence was gruped into 6 healthcare regions (Region 1: Skåne (Malmöhus and Kristianstads), Blekinge; Region 2: Halland, Västra Götaland (Älvsborg and Skaraborg), Värmland; Region 3: Kronoberg, Kalmar, Jönköping, Östergötland; Region 4: Stockholm, Gotland; Region 5: Uppsala, Sörmland, Västmanland, Dalarna (Kopparberg), Gävleborg, Örebro; Region 6: Jämtland, Västernorrland, Norrbotten, Västerbotten) | | | | |

## Figure 1. Flow chart of the patient selection

| Cancer surgery^1^ | Comparator^2^ |
| --- | --- |
| 454 589 | 4 234 248 |

|  | Cancer surgery^1^ | Comparator^2^ |
| --- | --- | --- |
| Incomplete date of death in the Cause of death register^6^ | 75 | 2 255 |
| Erroneous discharge date in the inpatient register | 5 | 46 |
| Missing county | 14 | 131 |

| Cancer surgery^1^ | Comparator^2^ |
| --- | --- |
| 454 495 | 4 231 816 |

|  | Cancer surgery^1^ | Comparator^2^ |
| --- | --- | --- |
| Index date before 1988 | 11 195 | 104 055 |
|  |  |  |

| Cancer surgery^1^ | Comparator^2^ |
| --- | --- |
| 443 300 | 4 127 761 |

| Cancer surgery^1^ | Comparator^2^ |
| --- | --- |
| 460 904 | 4 296 154 |

|  | Cancer surgery^1^ | Comparator^2^ |
| --- | --- | --- |
| Testicular cancer (all)^3^ | 5 752 | 56 698 |
| Kidney cancer^4^ | 335 | 3 059 |
| Gynecological cancer^5^ | 228 | 2 149 |

When a cancer patient was excluded, all matched population comparators were as well, special cases are further explained in notes:

^1^ All patients with selected cancers who were treated with surgery (see supplementary Table S1 for details on the inclusion criteria i.e., cancer and surgery codes).

^2^ Population comparators who were cancer free one year before the index date, matched on birth year, sex and county.

^3^ All patients with testicular cancer were excluded due to potential issues with data quality for this particular cancer cohort.

^4^ Patients with kidney cancer excluded if they had surgery code KAD52, KAD56, KAD98 or KAD60 recorded without at least one of the following codes: KAC20, KAD00, KAD01, KAB00, KAB01, JAH01, KAC00, PJD41, PJD42, PJD43, PJD44, PJD45, PJD51, PJD52, PJD53, PJD54, PJD55, PJD98 or PJD99. These patients were considered as not having a major surgery.

^5^ Patients with gynecological cancer excluded if they had surgery code LDB00 recorded without at least one of the following codes: JAH00, LAF10, LAF20, JAL30, LCD00, LCD10, PJD41, PJD42, PJD43, PJD44, PJD45, PJD51, PJD52, PJD53, PJD54, PJD55, PJD98 or PJD99. These patients were considered as not having a major surgery.

^6^ Incomplete date of death refers to date records having 00 as the day and/or 00 as the month of death. If a comparison subject had an incomplete date of death in the Cause of death register, only that subject was excluded. If a cancer surgery patient had an incomplete date of death in the Cause of death register, that subject and all matched comparison subjects were excluded. A subject who died on the day 00 had to survive at least 1 year + 1 month from the index date not to be excluded. A subject who had died on the month 00 had to survive for a minimum of 1 year from the end of the index year not to be excluded.

Further selection for analysis of the outcome after the discharge:

| Cancer surgery^1^ | Comparator^2^ |
| --- | --- |
| 443 300 | 4 127 761 |

|  | Cancer surgery^1^ | Comparator^2^ |
| --- | --- | --- |
| IS during hosp^6^ | 1 256 | 12 958 |
| Death from other causes during hosp^7^ | 5 229 | 48 810 |
| Index hosp ≥1 year | 7 | 62 |
|  |  |  |

|  | Cancer surgery^1^ | Comparator^2^ |
| --- | --- | --- |
| MI during hosp^6^ | 1 855 | 18 210 |
| Death from other causes during hosp^7^ | 4 735 | 44 272 |
| Index hosp ≥1 year | 7 | 63 |
|  |  |  |

| Cancer surgery^1^ | Comparator^2^ |
| --- | --- |
| 436 703 | 4 065 216 |

| Cancer surgery^1^ | Comparator^2^ |
| --- | --- |
| 436 808 | 4 065 931 |

Abbreviations: MI; Myocardial infarction; IS, Ischemic stroke; hosp, Hospitalization

^6^ If a comparison subject had an outcome during hospitalization, only that subject was excluded. If

A cancer surgery patient had an outcome during hospitalization, that subject and all matched comparison subjects were excluded.

^7^ If a comparison subject had died from causes other than outcome during hospitalization, only that subject was excluded, if a cancer surgery patient had died from causes other than outcome during hospitalization, that subject and all matched comparison subjects were excluded.

## Table 4. Patient characteristics for separate cancer types

|  | Bladder | Breast | CRC | Gallbl. | GE | Gyn. | Kidney | Lung | Pancr. | Prostate |  |
| --- | --- | --- | --- | --- | --- | --- | --- | --- | --- | --- | --- |
|  | % | % | % | % | % | % | % | % | % | % |  |
| Age (median) | 69 | 63 | 73 | 70 | 71 | 65 | 68 | 67 | 66 | 64 |  |
| Age groups |  |  |  |  |  |  |  |  |  |  |  |
| ≤49 | 3.79 | 17.56 | 4.44 | 4.54 | 5.92 | 15.84 | 9.02 | 7.16 | 8.96 | 1.53 |  |
| 50-59 | 12.38 | 22.39 | 9.98 | 13.50 | 13.46 | 19.49 | 15.82 | 16.67 | 17.72 | 21.02 |  |
| 60-69 | 35.42 | 27.44 | 23.43 | 30.83 | 26.92 | 28.14 | 29.98 | 37.86 | 35.27 | 60.69 |  |
| 70-79 | 40.35 | 20.42 | 35.63 | 34.32 | 36.83 | 25.44 | 33.62 | 34.24 | 33.74 | 16.65 |  |
| ≥80 | 8.06 | 12.19 | 26.53 | 16.81 | 16.87 | 11.08 | 11.55 | 4.07 | 4.31 | 0.11 |  |
| Sex, female | 24.30 | 99.46 | 48.52 | 67.45 | 35.20 | 100.00 | 40.55 | 47.32 | 49.60 | 0.00 |  |
| Hospitalization duration (median, days) | 17 | 2 | 11 | 12 | 16 | 7 | 8 | 7 | 17 | 3 |  |
| Outcome |  |  |  |  |  |  |  |  |  |  |  |
| Myocardial infarction, in total | 2.19 | 0.68 | 2.33 | 1.85 | 2.52 | 0.81 | 1.77 | 2.04 | 1.86 | 0.67 |  |
| during hospitalization | 0.81 | 0.06 | 0.97 | 0.66 | 1.25 | 0.20 | 0.53 | 0.49 | 0.82 | 0.07 |  |
| after discharge | 1.38 | 0.62 | 1.37 | 1.19 | 1.27 | 0.61 | 1.24 | 1.55 | 1.04 | 0.60 |  |
| Ischemic stroke, in total | 1.63 | 0.81 | 2.02 | 1.85 | 1.99 | 1.02 | 1.45 | 1.62 | 1.83 | 0.45 |  |
| during hospitalization | 0.45 | 0.09 | 0.58 | 0.40 | 0.67 | 0.22 | 0.34 | 0.45 | 0.27 | 0.04 |  |
| after discharge | 1.18 | 0.73 | 1.44 | 1.46 | 1.32 | 0.80 | 1.11 | 1.17 | 1.56 | 0.40 |  |
| Ischemic heart disease | 6.77 | 1.54 | 5.75 | 3.79 | 6.07 | 2.19 | 5.75 | 7.33 | 5.22 | 1.96 |  |
| History of myocardial infarction | 0.89 | 0.32 | 1.42 | 0.57 | 1.33 | 0.38 | 1.06 | 1.79 | 0.92 | 0.23 |  |
| Congestive Heart Failure | 1.87 | 1.05 | 3.42 | 2.07 | 2.50 | 1.19 | 2.97 | 2.06 | 1.36 | 0.38 |  |
| Cardiac Arrhythmia | 5.14 | 2.28 | 5.76 | 3.79 | 4.52 | 2.91 | 5.40 | 4.96 | 4.31 | 2.52 |  |
| Valvular disease | 1.07 | 0.49 | 1.23 | 0.49 | 1.03 | 0.53 | 1.27 | 1.08 | 0.87 | 0.58 |  |
| Peripheral Vascular Disease | 1.83 | 0.35 | 1.16 | 0.88 | 1.41 | 0.30 | 1.85 | 2.77 | 1.24 | 0.36 |  |
| Hypertension | 18.37 | 4.20 | 9.73 | 9.62 | 9.05 | 8.17 | 13.57 | 11.40 | 16.88 | 5.89 |  |
| Cerebrovascular Disease | 1.75 | 0.98 | 2.66 | 1.76 | 2.04 | 1.23 | 2.21 | 2.03 | 1.51 | 0.68 |  |
| History of ischemic stroke | 1.02 | 0.58 | 1.66 | 1.10 | 1.30 | 0.75 | 1.17 | 1.19 | 0.72 | 0.27 |  |
| TIA | 0.31 | 0.22 | 0.54 | 0.31 | 0.41 | 0.27 | 0.51 | 0.53 | 0.32 | 0.24 |  |
| Chronic Pulmonary Disease | 4.77 | 1.41 | 2.71 | 2.51 | 3.07 | 1.54 | 3.07 | 12.21 | 4.02 | 0.89 |  |
| Diabetes Mellitus | 7.14 | 2.10 | 5.76 | 6.04 | 5.24 | 3.91 | 6.17 | 5.34 | 15.02 | 2.10 |  |
| Renal Disease | 1.37 | 0.25 | 0.77 | 0.13 | 0.59 | 0.31 | 1.92 | 0.72 | 0.59 | 0.33 |  |
| Anemia | 0.76 | 0.24 | 7.35 | 0.97 | 4.62 | 0.93 | 1.25 | 0.55 | 0.69 | 0.11 |  |
| All-cause mortality (at 90 days) | 4.67 | 0.53 | 5.84 | 17.16 | 9.37 | 2.21 | 3.76 | 3.45 | 6.66 | 0.16 |  |
| All-cause mortality (at 1 year) | 20.95 | 2.89 | 16.13 | 52.40 | 33.71 | 8.44 | 13.71 | 14.98 | 33.99 | 0.51 |  |
| Abbreviations: CRC, Colorectal; Gallbl. Gallbladder; GE. Gastroesophageal; Gyn. Gynecological; Pancr. Pancreatic; TIA. Transient ischemic attack | | | | | | | | | | | |

## Table 5. Crude ORs of arterial ischemic events during the first year of follow up

| Cancer type |  | **OR** | **95 % CIs** |
| --- | --- | --- | --- |
| All cancers | MI | 1.28 | 1.25, 1.32 |
|  | IS | 1.25 | 1.22, 1.29 |
| Bladder | MI | 1.65 | 1.41, 1.93 |
|  | IS | 1.46 | 1.22, 1.74 |
| Breast | MI | 1.01 | 0.95, 1.08 |
|  | IS | 1.09 | 1.03, 1.16 |
| Colorectal | MI | 1.40 | 1.34, 1.45 |
|  | IS | 1.30 | 1.24, 1.36 |
| Gallbladder | MI | 1.42 | 1.02, 1.97 |
|  | IS | 1.69 | 1.21, 2.35 |
| Gastroesophageal | MI | 1.56 | 1.39, 1.73 |
|  | IS | 1.48 | 1.31, 1.68 |
| Gynecological | MI | 1.16 | 1.05, 1.27 |
|  | IS | 1.37 | 1.26, 1.50 |
| Kidney and UTUC | MI | 1.51 | 1.36, 1.69 |
|  | IS | 1.35 | 1.20, 1.52 |
| Lung | MI | 2.02 | 1.77, 2.29 |
|  | IS | 1.89 | 1.64, 2.18 |
| Pancreatic | MI | 2.09 | 1.62, 2.63 |
|  | IS | 2.17 | 1.68, 2.80 |
| Prostate | MI | 0.71 | 0.62, 0.80 |
|  | IS | 0.63 | 0.54, 0.73 |

## Figure 2. Hazard ratios for myocardial infarction and ischemic stroke after discharge from the hospital

## Figure 3. Cumulative incidence function for myocardial infarction after discharge from the hospital


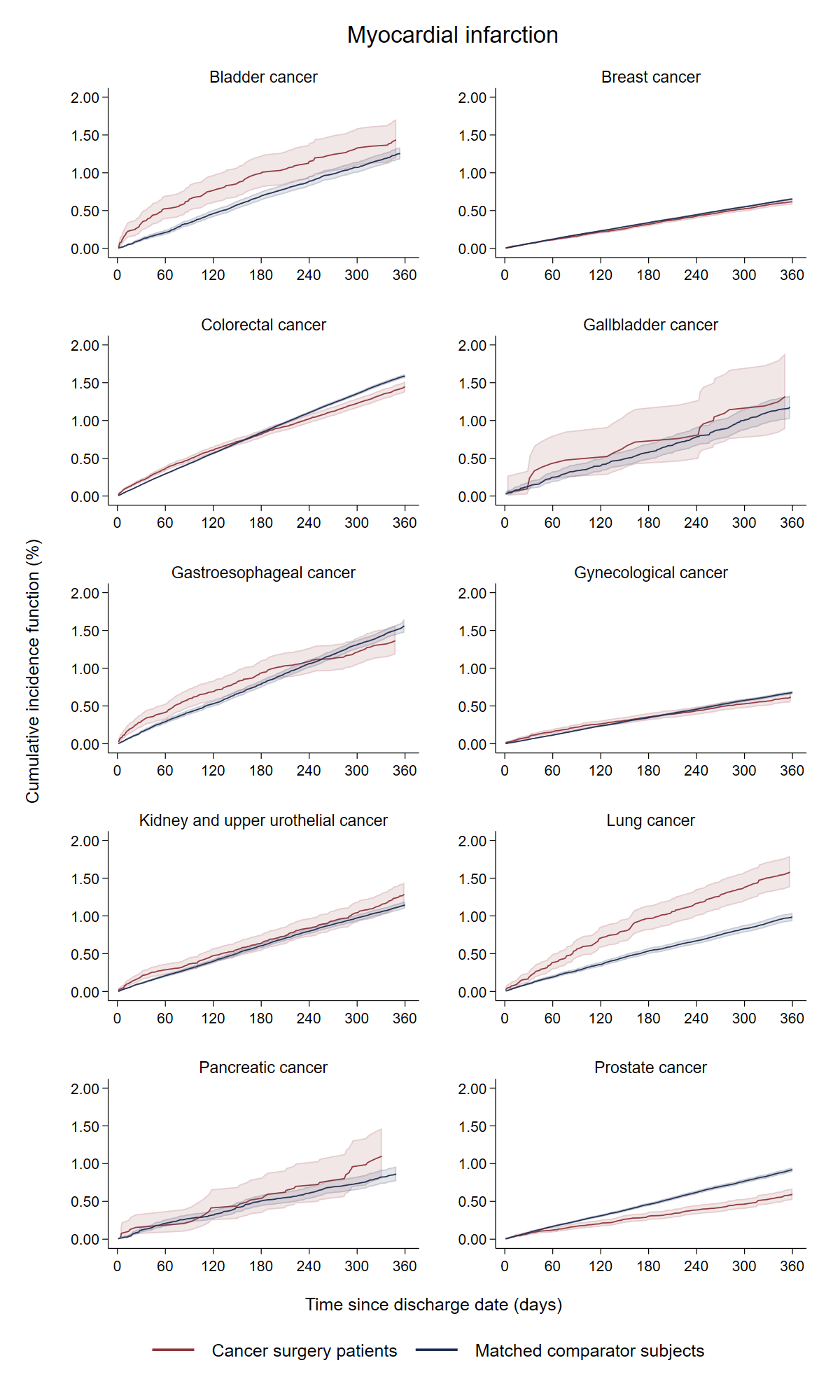


## Figure 4. Cumulative incidence function for ischemic stroke after discharge from the hospital


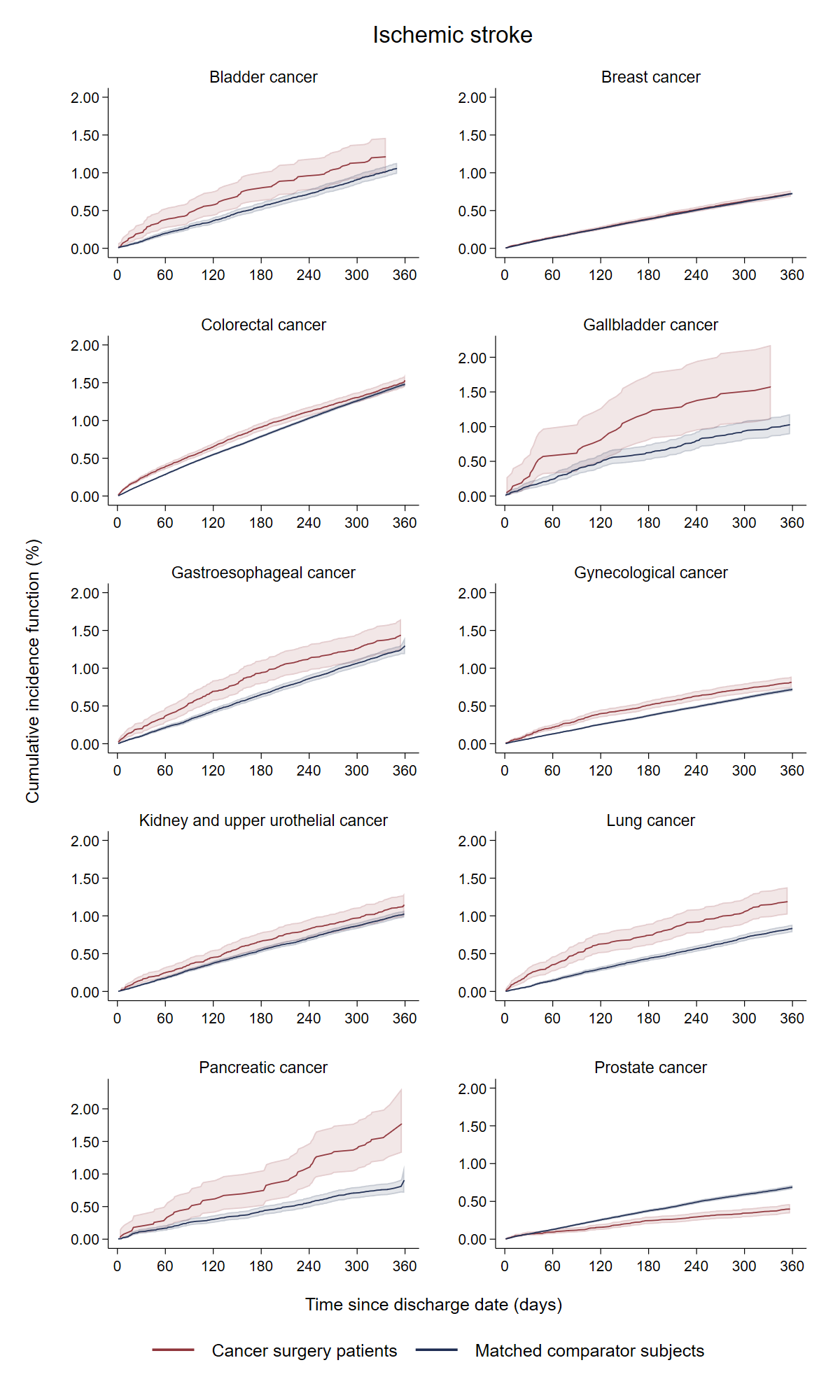


## Table 6. Lethal or severe myocardial infarctions during the first postoperative year

| **Cancer** | **Outcome** | **Subject** | **Outcome (one year after index date)** | | | | | **Post-discharge outcome** | | | | |
| --- | --- | --- | --- | --- | --- | --- | --- | --- | --- | --- | --- | --- |
|  |  |  | **N** | **Cause of death^1^** | | **Procedure^2^** | | **N** | **Cause of death** | | **Procedure^2^** | |
|  |  |  |  | **N** | **%** | **N** | **%** |  | **N** | **%** | **N** | **%** |
| Bladder | MI | Comparators | 1 035 | 245 | 23.67 | 136 | 13.14 | 925 | 216 | 23.35 | 130 | 14.05 |
|  |  | Cases | 188 | 27 | 14.36 | 10 | 5.32 | 119 | 25 | 21.01 | 7 | 5.88 |
|  | IS | Comparators | 878 | 78 | 8.88 | 2 | 0.23 | 789 | 68 | 8.62 | 1 | 0.13 |
|  |  | Cases | 136 | 10 | 7.35 | 0 | 0.00 | 98 | 8 | 8.16 | 0 | 0.00 |
| Breast | MI | Comparators | 10 141 | 2 238 | 22.07 | 917 | 9.04 | 9 925 | 2 181 | 21.97 | 907 | 9.14 |
|  |  | Cases | 1 078 | 209 | 19.39 | 83 | 7.70 | 979 | 206 | 21.04 | 83 | 8.48 |
|  | IS | Comparators | 11 286 | 1 421 | 12.59 | 17 | 0.15 | 11 031 | 1 374 | 12.46 | 17 | 0.15 |
|  |  | Cases | 1 311 | 77 | 5.87 | 1 | 0.08 | 1 169 | 76 | 6.50 | 1 | 0.09 |
| Colorectal | MI | Comparators | 17 259 | 4 306 | 24.95 | 1 427 | 8.27 | 15 432 | 3 803 | 24.64 | 1 325 | 8.59 |
|  |  | Cases | 2 641 | 482 | 18.25 | 108 | 4.09 | 1 536 | 361 | 23.50 | 104 | 6.77 |
|  | IS | Comparators | 16 005 | 2 091 | 13.06 | 36 | 0.22 | 14 349 | 1 819 | 12.68 | 36 | 0.25 |
|  |  | Cases | 2 269 | 179 | 7.89 | 2 | 0.09 | 1 603 | 151 | 9.42 | 2 | 0.12 |
| Gallbladder | MI | Comparators | 266 | 63 | 23.68 | 11 | 4.14 | 214 | 53 | 24.77 | 11 | 5.14 |
|  |  | Cases | 42 | 8 | 19.05 | 0 | 0.00 | 27 | 7 | 25.93 | 0 | 0.00 |
|  | IS | Comparators | 224 | 26 | 11.61 | 0 | 0.00 | 190 | 22 | 11.58 | 0 | 0.00 |
|  |  | Cases | 42 | 4 | 9.52 | 0 | 0.00 | 33 | 4 | 12.12 | 0 | 0.00 |
| Gynecological | MI | Comparators | 3 794 | 795 | 20.95 | 334 | 8.80 | 3 592 | 746 | 20.77 | 317 | 8.83 |
|  |  | Cases | 461 | 89 | 19.31 | 21 | 4.56 | 344 | 83 | 24.13 | 21 | 6.10 |
|  | IS | Comparators | 4 034 | 447 | 11.08 | 5 | 0.12 | 3 830 | 416 | 10.86 | 5 | 0.13 |
|  |  | Cases | 588 | 40 | 6.80 | 2 | 0.34 | 463 | 38 | 8.21 | 2 | 0.43 |
| Kidney | MI | Comparators | 2 283 | 517 | 22.65 | 272 | 11.91 | 2 152 | 485 | 22.54 | 264 | 12.27 |
|  |  | Cases | 372 | 47 | 12.63 | 29 | 7.80 | 260 | 38 | 14.62 | 29 | 11.15 |
|  | IS | Comparators | 2 071 | 212 | 10.24 | 7 | 0.34 | 1 918 | 193 | 10.06 | 7 | 0.36 |
|  |  | Cases | 301 | 11 | 3.65 | 1 | 0.33 | 229 | 9 | 3.93 | 1 | 0.44 |
| Lung | MI | Comparators | 1 318 | 287 | 21.78 | 169 | 12.82 | 1 242 | 265 | 21.34 | 162 | 13.04 |
|  |  | Cases | 284 | 53 | 18.66 | 26 | 9.15 | 215 | 45 | 20.93 | 24 | 11.16 |
|  | IS | Comparators | 1 112 | 94 | 8.45 | 4 | 0.36 | 1 050 | 87 | 8.29 | 4 | 0.38 |
|  |  | Cases | 222 | 7 | 3.15 | 1 | 0.45 | 159 | 7 | 4.40 | 0 | 0.00 |
| Pancreas | MI | Comparators | 328 | 59 | 17.99 | 50 | 15.24 | 294 | 48 | 16.33 | 45 | 15.31 |
|  |  | Cases | 75 | 13 | 17.33 | 5 | 6.67 | 42 | 9 | 21.43 | 4 | 9.52 |
|  | IS | Comparators | 314 | 24 | 7.64 | 0 | 0.00 | 274 | 22 | 8.03 | 0 | 0.00 |
|  |  | Cases | 72 | 10 | 13.89 | 0 | 0.00 | 62 | 8 | 12.90 | 0 | 0.00 |
| Prostate | MI | Comparators | 3 498 | 616 | 17.61 | 1 028 | 29.39 | 3 428 | 604 | 17.62 | 1 009 | 29.43 |
|  |  | Cases | 264 | 21 | 7.95 | 98 | 37.12 | 236 | 21 | 8.90 | 97 | 41.10 |
|  | IS | Comparators | 2 615 | 115 | 4.40 | 12 | 0.46 | 2 550 | 107 | 4.20 | 12 | 0.47 |
|  |  | Cases | 176 | 0 | 0.00 | 1 | 0.57 | 158 | 0 | 0.00 | 1 | 0.63 |
| Gastroesophageal | MI | Comparators | 2 228 | 527 | 23.65 | 163 | 7.32 | 1 908 | 434 | 22.75 | 152 | 7.97 |
|  |  | Cases | 379 | 93 | 24.54 | 8 | 2.11 | 188 | 61 | 32.45 | 6 | 3.19 |
|  | IS | Comparators | 1 853 | 196 | 10.58 | 4 | 0.22 | 1 560 | 156 | 10.00 | 4 | 0.26 |
|  |  | Cases | 301 | 35 | 11.63 | 0 | 0.00 | 198 | 27 | 13.64 | 0 | 0.00 |
| Abbreviations: MI, Myocardial infarction; IS, Ischemic stroke; COD, The Swedish cause of death register;  ^1^ Outcomes identified from the cause of death register, where cause of death from interest was either main or any of the contributory causes of death  ^2^ Outcomes identified from the National Patient register, where in addition to outcome codes a patient also had a record of following coronary surgery-related procedural codes: ICD-9: 3065-66, 3068, 3080, 3092, 3105, 3127, 3158 or IDC-10: FN* | | | | | | | | | | | | |

## Table 7. Baseline characteristics of cancer surgery population and comparison population, sensitivity analysis of patients recruited since 2002

| Cancer type | Cancer surgery population  (N=255 819) | | Comparison population  (N=2 378 404) | |
| --- | --- | --- | --- | --- |
|  | n (%) |  | n (%) |  |
| Bladder | 5 377 (2.10) | | 48 675 (2.05) | |
| Breast | 93 804 (36.67) | | 887 724 (37.32) | |
| Colorectal | 61 859 (24.18) | | 557 654 (23.45) | |
| Gallbladder | 867 (0.34) | | 8 001 (0.34) | |
| Gastroesophageal | 6 007 (2.35) | | 54 959 (2.31) | |
| Gynecological | 30 348 (11.86) | | 285 534 (12.01) | |
| Kidney and upper urothelial | 12 000 (4.69) | | 110 522 (4.65) | |
| Lung | 8 048 (3.15) | | 74 601 (3.14) | |
| Pancreatic | 2 610 (1.02) | | 24 170 (1.02) | |
| Prostate | 34 899 (13.64) | | 326 564 (13.73) | |
| Age (median, IQR) | 67 (59, 74) | | 66 (58, 74) | |
| Age groups |  |  |  |  |
| ≤49 | 25 400 (9.93) | | 250 835 (10.55) | |
| 50-59 | 43 717 (17.09) | | 425 082 (17.87) | |
| 60-69 | 84 947 (33.21) | | 800 302 (33.65) | |
| 70-79 | 65 725 (25.69) | | 592 004 (24.89) | |
| ≥80 | 36 030 (14.08) | | 310 181 (13.04) | |
| Women | 167 397 (65.44) | | 1 574 048 (66.18) | |
| Length of hospitalization (days, median, IQR) | 4 (2.00, 8.00) | |  |  |
| Outcome |  |  |  |  |
| Myocardial infarction, in total | 2 976 (1.16) | | 21 296 (0.90) | |
| Myocardial infarction, during hospitalization | 911 (0.36) | | 720 (0.03) | |
| Myocardial infarction, after discharge | 2 065 (0.81) | | 20 576 (0.87) | |
| Ischemic stroke, in total | 2 882 (1.13) | | 21 465 (0.90) | |
| Ischemic stroke, during hospitalization | 572 (0.22) | | 747 (0.03) | |
| Ischemic stroke, after discharge | 2 310 (0.90) | | 20 718 (0.87) | |
| Ischemic heart disease | 10 775 (4.21) | | 68 064 (2.86) | |
| History of myocardial infarction | 1 739 (0.68) | | 13 745 (0.58) | |
| Congestive Heart Failure | 5 080 (1.99) | | 36 892 (1.55) | |
| Cardiac Arrhythmia | 12 761 (4.99) | | 77 336 (3.25) | |
| Valvular disease | 2 768 (1.08) | | 16 137 (0.68) | |
| Peripheral Vascular Disease | 2 669 (1.04) | | 16 633 (0.70) | |
| Hypertension | 29 527 (11.54) | | 130 889 (5.50) | |
| Cerebrovascular Disease | 4 651 (1.82) | | 39 085 (1.64) | |
| History of ischemic stroke | 2 597 (1.02) | | 22 967 (0.97) | |
| TIA | 1 024 (0.40) | | 7 998 (0.34) | |
| Chronic Pulmonary Disease | 7 977 (3.12) | | 41 510 (1.75) | |
| Diabetes Mellitus | 13 269 (5.19) | | 78 957 (3.32) | |
| Renal Disease | 1 981 (0.77) | | 13 535 (0.57) | |
| Anemia | 7 138 (2.79) | | 7 237 (0.30) | |
| All-cause mortality, at 90 days | 5 643 (2.21) | | 11 698 (0.49) | |
| All-cause mortality, at 1 year | 18 699 (7.31) | | 49 178 (2.07) | |
| Abbreviations: IQR, Interquartile range; NA, Not applicable | | | | |

## Table 8. Absolute risks for arterial ischemic events during the first year of follow up, sensitivity analysis of patients recruited since 2002.

| Cancer type |  | Absolute risk | | Absolute risk difference | | |
| --- | --- | --- | --- | --- | --- | --- |
|  |  | Cancer surgery population (%) | Comparison population (%) | % | | 95% CIs |
| All cancers | MI | 1.16 | 0.90 | 0.27 | 0.22, 0.31 | |
|  | IS | 1.13 | 0.90 | 0.22 | 0.18, 0.27 | |
| Bladder | MI | 2.12 | 1.18 | 0.94 | 0.54, 1.34 | |
|  | IS | 1.88 | 1.11 | 0.77 | 0.39, 1.14 | |
| Breast | MI | 0.54 | 0.58 | -0.04 | -0.09, 0.01 | |
|  | IS | 0.72 | 0.68 | 0.04 | -0.02, 0.10 | |
| Colorectal | MI | 2.25 | 1.43 | 0.82 | 0.70, 0.94 | |
|  | IS | 1.90 | 1.41 | 0.50 | 0.38, 0.61 | |
| Gallbladder | MI | 1.61 | 0.92 | 0.69 | -0.17, 1.55 | |
|  | IS | 2.65 | 0.85 | 1.80 | 0.71, 2.89 | |
| Gastroesophageal | MI | 2.03 | 1.29 | 0.74 | 0.37, 1.11 | |
|  | IS | 1.73 | 1.14 | 0.59 | 0.25, 0.93 | |
| Gynecological | MI | 0.80 | 0.68 | 0.12 | 0.02, 0.22 | |
|  | IS | 1.07 | 0.77 | 0.30 | 0.18, 0.42 | |
| Kidney and UTUC | MI | 1.50 | 0.99 | 0.51 | 0.28, 0.73 | |
|  | IS | 1.42 | 0.96 | 0.45 | 0.23, 0.67 | |
| Lung | MI | 1.74 | 0.84 | 0.90 | 0.60, 1.19 | |
|  | IS | 1.52 | 0.83 | 0.68 | 0.41, 0.96 | |
| Pancreatic | MI | 1.61 | 0.80 | 0.81 | 0.32, 1.31 | |
|  | IS | 1.65 | 0.89 | 0.76 | 0.26, 1.26 | |
| Prostate | MI | 0.64 | 0.90 | -0.26 | -0.35, -0.17 | |
|  | IS | 0.41 | 0.69 | -0.28 | -0.35, -0.21 | |
| Abbreviations: CIs, confidence intervals; MI, Myocardial infarction; IS, Ischemic stroke; UTUC, Upper urothelial cancer | | | | | | |

## Table 9. Risk of arterial ischemic events during hospitalization in cancer surgery population compared to matched comparison population, sensitivity analysis of patients recruited since 2002

| Cancer type | Myocardial infarction | | | | | Ischemic stroke | | | | | | | |
| --- | --- | --- | --- | --- | --- | --- | --- | --- | --- | --- | --- | --- | --- |
|  | OR | 95% CIs | | | | OR | | | 95% CIs | | | | |
| Bladder | 9.02 | 5.80, 14.04 | | | | | 6.48 | | | 3.96, 10.62 | | | |
| Breast | 4.49 | 3.01, 6.69 | | | | | 5.49 | | | 3.90, 7.72 | | | |
| Colorectal | 12.91 | 11.36, 14.68 | | | | | 7.11 | | | 6.10, 8.28 | | | |
| Gallbladder | 10.31 | 3.71, 28.60 | | | | | 6.43 | | | 2.01, 20.60 | | | |
| Gastroesophageal | 13.69 | 9.33, 20.08 | | | | | 5.34 | | | 3.38, 8.43 | | | |
| Gynecological | 8.65 | 5.99, 12.48 | | | | | 9.46 | | | 6.64, 13.47 | | | |
| Kidney and UTUC | 12.07 | 7.84, 18.60 | | | | | 6.60 | | | 4.30, 10.12 | | | |
| Lung | 23.21 | 11.55, 46.64 | | | | | 18.97 | | | 10.23, 35.18 | | | |
| Pancreas | 13.25 | 6.52, 26.90 | | | | | 5.19 | | | 2.16, 12.42 | | | |
| Prostate | 5.25 | 3.21, 8.58 | | | | | 2.48 | | | 1.37, 4.49 | | | |
| All cancers | 11.18 | 10.14, 12.33 | | | | | 6.80 | | | 6.10, 7.59 | | | |
| Women | 0.36 | 0.32, 0.39 | | | | | 0.52 | | | 0.47, 0.58 | | | |
| Age groups |  |  |  | |  | | |  | | |  | |  |
| ≤49 | 0.14 | 0.07, 0.29 | | | | | 0.17 | | | 0.09, 0.32 | | | |
| 50-59 | 0.35 | 0.25, 0.48 | | | | | 0.45 | | | 0.33, 0.62 | | | |
| 60-69 | 1.00 |  | |  | | | 1.00 | | |  | |  | |
| 70-79 | 3.08 | 2.66, 3.56 | | | | | 3.06 | | | 2.61, 3.59 | | | |
| ≥80 | 8.26 | 7.17, 9.52 | | | | | 7.04 | | | 6.02, 8.23 | | | |
| Abbreviations: OR, Odds ratio; CIs, Confidence intervals; UTUC, Upper urothelial cancer | | | | | | | | | | | | | |

## Table 10. Risk of arterial ischemic events after discharge from the hospital in the cancer surgery population compared to comparison population, sensitivity analysis of patients recruited since 2002.

| Cancer type | Time | Myocardial infarction | | | Ischemic stroke | | |
| --- | --- | --- | --- | --- | --- | --- | --- |
|  |  | HR | 95% CIs | | HR | 95% CIs | |
| Bladder | Average | 1.16 | 0.90, 1.48 | | 1.35 | 1.05, 1.73 | |
|  | 30d | 2.13 | 1.38, 3.29 | | 2.18 | 1.36, 3.51 | |
|  | 90d | 1.07 | 0.73, 1.58 | | 1.52 | 1.05, 2.20 | |
|  | 1y | 1.01 | 0.58, 1.77 | | 0.90 | 0.47, 1.72 | |
| Breast | Average | 0.84 | 0.77, 0.93 | | 0.97 | 0.89, 1.05 | |
|  | 30d | 0.85 | 0.69, 1.05 | | 1.13 | 0.94, 1.34 | |
|  | 90d | 0.82 | 0.72, 0.95 | | 1.02 | 0.90, 1.15 | |
|  | 1y | 0.82 | 0.67, 1.01 | | 0.85 | 0.70, 1.03 | |
| Colorectal | Average | 0.92 | 0.85, 0.99 | | 1.04 | 0.97, 1.12 | |
|  | 30d | 1.18 | 1.02, 1.37 | | 1.37 | 1.19, 1.57 | |
|  | 90d | 0.86 | 0.76, 0.96 | | 1.03 | 0.92, 1.15 | |
|  | 1y | 0.88 | 0.75, 1.04 | | 0.88 | 0.75, 1.04 | |
| Gallbladder | Average | 0.90 | 0.38, 2.16 | | 2.65 | 1.49, 4.72 | |
|  | 30d | 1.39 | 0.28, 7.03 | | 2.68 | 1.02, 7.08 | |
|  | 90d | 0.88 | 0.15, 5.29 | | 1.94 | 0.61, 6.10 | |
|  | 1y | 1.17 | 0.18, 7.36 | | 2.96 | 0.92, 9.53 | |
| Gastroesophageal | Average | 0.73 | 0.55, 0.97 | | 1.37 | 1.07, 1.76 | |
|  | 30d | 0.72 | 0.42, 1.25 | | 1.75 | 1.07, 2.89 | |
|  | 90d | 0.56 | 0.34, 0.91 | | 1.58 | 1.13, 2.20 | |
|  | 1y | 0.84 | 0.44, 1.59 | | 1.08 | 0.61, 1.90 | |
| Gynecological | Average | 0.87 | 0.75, 1.02 | | 1.12 | 0.99, 1.28 | |
|  | 30d | 1.21 | 0.89, 1.65 | | 1.79 | 1.39, 2.31 | |
|  | 90d | 0.86 | 0.69, 1.08 | | 1.27 | 1.05, 1.53 | |
|  | 1y | 0.74 | 0.52, 1.05 | | 0.76 | 0.55, 1.04 | |
| Kidney and UTUC | Average | 0.97 | 0.80, 1.16 | | 1.12 | 0.93, 1.35 | |
|  | 30d | 0.99 | 0.68, 1.46 | | 1.27 | 0.88, 1.84 | |
|  | 90d | 0.81 | 0.61, 1.07 | | 1.17 | 0.90, 1.52 | |
|  | 1y | 1.15 | 0.79, 1.69 | | 0.98 | 0.64, 1.51 | |
| Lung | Average | 1.42 | 1.15, 1.76 | | 1.30 | 1.03, 1.63 | |
|  | 30d | 1.49 | 0.98, 2.26 | | 2.23 | 1.46, 3.39 | |
|  | 90d | 1.43 | 1.06, 1.93 | | 1.34 | 0.96, 1.88 | |
|  | 1y | 1.30 | 0.82, 2.06 | | 0.81 | 0.46, 1.44 | |
| Pancreatic | Average | 1.11 | 0.71, 1.75 | | 1.70 | 1.17, 2.48 | |
|  | 30d | 0.54 | 0.20, 1.47 | | 1.71 | 0.76, 3.83 | |
|  | 90d | 0.91 | 0.47, 1.77 | | 1.23 | 0.63, 2.38 | |
|  | 1y | 1.64 | 0.64, 4.22 | | 3.14 | 1.49, 6.64 | |
| Prostate | Average | 0.69 | 0.60, 0.80 | | 0.64 | 0.54, 0.76 | |
|  | 30d | 0.76 | 0.55, 1.06 | | 0.77 | 0.54, 1.08 | |
|  | 90d | 0.64 | 0.52, 0.81 | | 0.66 | 0.51, 0.85 | |
|  | 1y | 0.76 | 0.57, 1.02 | | 0.51 | 0.33, 0.78 | |
| All cancers | Average | 0.87 | 0.84, 0.92 | | 1.01 | 0.97, 1.05 | |
|  | 30d | 1.05 | 0.95, 1.15 | | 1.32 | 1.21, 1.44 | |
|  | 90d | 0.83 | 0.77, 0.89 | | 1.05 | 0.98, 1.12 | |
|  | 1y | 0.86 | 0.78, 0.95 | | 0.83 | 0.75, 0.92 | |
| Female |  | 0.58 | 0.56, 0.60 | | 0.79 | 0.77, 0.81 | |
| Age groups |  |  |  |  |  |  |  |
| ≤49 |  | 0.12 | 0.10, 0.14 | | 0.13 | 0.11, 0.15 | |
| 50-59 |  | 0.45 | 0.43, 0.48 | | 0.43 | 0.40, 0.46 | |
| 60-69 |  | 1.00 |  |  | 1.00 |  |  |
| 70-79 |  | 1.79 | 1.73, 1.85 | | 2.11 | 2.04, 2.19 | |
| ≥80 |  | 3.93 | 3.79, 4.07 | | 4.52 | 4.36, 4.69 | |
| Abbreviations: MI, Myocardial infarction; IS, Ischemic stroke; HR, Hazard ratio; CIs, Confidence intervals; d, Day; y, Year; UTUC, Upper tract urothelial cancer | | | | | | | |

## Figure 5. Cumulative incidence of myocardial infarction and ischemic stroke after discharge from the hospital, patients after 2002

## Figure 6. Hazard ratios for myocardial infarction and ischemic stroke after discharge from the hospital, patients after 2002

## Figure legends

Figure 1. Flow chart of the selection of the study population

Figure 2. The figure presents hazard ratios for myocardial infarction and ischemic stroke in the entire cancer cohort after discharge from the hospital in relation to matched comparator subjects.

Figure 3. The figure illustrates the cumulative incidence function of myocardial infarction among patients operated for major cancer surgery.

Figure 4. The figure illustrates the cumulative incidence function of ischemic stroke among patients operated for major cancer surgery after the discharge from the hospital.

Figure 5. The figure illustrates the cumulative incidence of myocardial infarction and ischemic stroke after discharge from hospital after the index oncological surgery. A sensitivity analysis for patients recruited after 2002.

Figure 6. The figure presents hazard ratios for myocardial infarction for different cancer types after discharge from the hospital in relation to matched comparator subjects. A sensitivity analysis for patients recruited after 2002.
